# Mitochondrial Protein *Akap*1 Deletion Exacerbates Endoplasmic Reticulum Stress in Mice Exposed to Hyperoxia

**DOI:** 10.3389/fphar.2022.762840

**Published:** 2022-03-14

**Authors:** Sahebgowda Sidramagowda Patil, Ramani Soundararajan, Jutaro Fukumoto, Mason Breitzig, Helena Hernández-Cuervo, Matthew Alleyn, Muling Lin, Venkata Ramireddy Narala, Richard Lockey, Narasaiah Kolliputi, Lakshmi Galam

**Affiliations:** ^1^ University of South Florida, Division of Allergy and Immunology, Department of Internal Medicine, College of Medicine, Tampa, FL, United States; ^2^ Washington University in St. Louis, Brown School, St. Louis, MO, United States; ^3^ University of South Florida, Department of Molecular Medicine, College of Medicine, Tampa, FL, United States; ^4^ Department of Zoology, Yogi Vemana University, Kadapa, India

**Keywords:** ALI, ARDS, ROS, ER stress, Akap1

## Abstract

Acute lung injury (ALI) and its severe manifestation, acute respiratory distress syndrome (ARDS), are treated with high concentrations of supplementary oxygen. However, prolonged exposure to high oxygen concentrations stimulates the production of reactive oxygen species (ROS), which damages the mitochondria and accumulates misfolded proteins in the endoplasmic reticulum (ER). The mitochondrial protein A-kinase anchoring protein 1 (*Akap1*) is critical for mitochondrial homeostasis. It is known that *Akap1* deficiency results in heart damage, neuronal development impairment, and mitochondrial malfunction in preclinical studies. Our laboratory recently revealed that deleting *Akap1* increases the severity of hyperoxia-induced ALI in mice. To assess the role of *Akap1* deletion in ER stress in lung injury, wild-type and *Akap1* ^*−/−*^ mice were exposed to hyperoxia for 48 h. This study indicates that *Akap1* ^*−/−*^ mice exposed to hyperoxia undergo ER stress, which is associated with an increased expression of BiP, JNK phosphorylation, eIF2α phosphorylation, ER stress-induced cell death, and autophagy. This work demonstrates that deleting *Akap1* results in increased ER stress in the lungs of mice and that hyperoxia exacerbates ER stress-related consequences.

## Introduction

The most common treatment for human acute respiratory distress syndrome is supplemental oxygen (Kallet and Matthay, 2013). However, prolonged exposure to high concentrations of supplementary oxygen leads to increased production of reactive oxygen species (ROS). This prolonged exposure induces hyperoxic acute lung injury in rodent models (Galam et al., 2015), leading to death (Kwak et al., 2006; Kallet and Matthay, 2013). This hyperoxia-induced stress (Fukumoto et al., 2013) can cause protein misfolding in the ER and trigger unfolded protein response (UPR) (Gewandter et al., 2009). The endoplasmic reticulum (ER) possesses receptors to alleviate stress by activation of PERK (protein kinase-like ER kinase), activation of transcription factor 6 (ATF6), and inositol requiring enzyme 1 (IRE1α) (Gewandter et al., 2009). The binding immunoglobulin protein (BiP) also promotes proper folding of proteins (Sano and Reed, 2013). ER stress leads to C-junction N-terminal kinase (JNK) phosphorylation, eukaryotic initiation factor (eIF2α) phosphorylation, ER stress-induced cell death, and autophagy (Sano and Reed, 2013; Lee et al., 2015; Hoffman et al., 2016; Cnop et al., 2017). The rodent hyperoxia model mimics the clinical presentation of ALI by augmenting oxidative stress (Kallet and Matthay, 2013) and exacerbating respiratory failure through ER stress, events for which there are no viable treatments (Gewandter et al., 2009). Therefore, investigating the pathways and mechanisms of acute lung injury in rodents caused by hyperoxia is crucial to finding an effective treatment for acute lung injury in humans. Likewise, the mechanistic role of mitochondrial proteins in hyperoxia-induced ALI needs clarification.

Mitochondria play an essential role in oxidative metabolism, ROS generation, cell cycle progression, and other key biological pathways (McBride et al., 2006; Nagar et al., 2018). Mitochondrial integrity is affected by hyperoxia as it enhances ROS production and thus the levels of lipid peroxidation byproducts, including the reactive aldehyde 4-hydroxy-2-nonenal (4-HNE) (Kolliputi and Waxman, 2009a; Galam et al., 2015; Breitzig et al., 2016; Narala et al., 2018). The resultant mitochondrial disturbances are associated with ER stress due to cross talk between mitochondria and the ER (Malhotra and Kaufman, 2011; van Vliet and Agostinis, 2018; Chu et al., 2019). ER stress is also associated with neurodegenerative diseases, ophthalmological disorders, cancer, inflammation, and metabolic diseases (Sano and Reed, 2013). The cross-link between mitochondria and the ER suggests that targeting ER stress can offer novel insights into the treatment of acute lung injury.

Within mitochondria, *Akap1*, a scaffolding protein, plays an important role in PKA regulation, cAMP signaling, and maintenance of mitochondrial homeostasis (Carlucci et al., 2008a; Merrill and Strack, 2014). *Akap*1 is abundantly expressed in the liver, heart, brain, kidney, and skeletal muscles and has three isoforms: AKAP121 (mouse), AKAP149 (human), and AKAP84 (alternative splicing) (Merrill and Strack, 2014). The downregulation of *Akap1* causes mitochondrial damage, cardiac dysfunction, lung injury, and neuronal death (Perrino et al., 2010; Merrill and Strack, 2014; Narala et al., 2018). *Akap1* deletion is associated with autophagy, mitophagy, and apoptosis in murine studies (Merrill and Strack, 2014; Narala et al., 2018). The significance of *Akap1* has been extensively studied in hypoxia-induced cardiac disease. Our laboratory has studied the role of *Akap1* in hyperoxic lung injury. However, the role of *Akap1* concerning ER stress during hyperoxia is unknown. It is hypothesized that *Akap1* deletion will exacerbate ER stress associated with hyperoxia.

## Materials and Methods

### Mice

All animal procedures were approved by the Institutional Animal Care and Use Committee of the University of South Florida. Dr. Stanley McKnight (University of Washington) generated the *Akap1* ^*−/−*^ mice (Newhall et al., 2006) and donated them to Dr. Stefan Strack (The University of Iowa). The *Akap1* ^*−/−*^ mice were generously donated by Dr. Stefan Strack. All the mice aged 7–9 weeks were accommodated in individually ventilated cages and maintained under similar conditions of a dark–light cycle, humidity (60 ± 5%), and temperature (22 ± 1°C). The mice were fed a regular diet *ad libitum*.

### Genotyping

The *Akap1* genotype was determined by PCR, and primers were used according to a previous study (Flippo et al., 2018).

### *In Vivo* Hyperoxia Exposure

Wild-type (*Wt*) and *Akap1* ^*−/−*^ mice were kept in cages within an airtight hyperoxia cabinet (75 × 50 × 50 cm) and exposed to 100% oxygen for 48 h in a specific pathogen-free environment. The oxygen concentration was measured by using a proOx p100 sensor (Biospherix, New York, NY) as described previously (Kolliputi and Waxman, 2009a).

### Quantitative Real-Time PCR

*Wt* and *Akap1* ^*−/−*^ mice lungs were collected after normoxia and hyperoxia (48 h) exposure. Total RNA was extracted from the lungs by using RNeasy kit (Qiagen, Hilden, Germany) and reverse-transcribed by using the iScript cDNA synthesis kit (Biorad Laboratories, Hercules, CA) and 1 ug of total RNA was used. qRT-PCR was performed for PERK, IRE1α, and ATF6 by using the SsoFast EvaGreen Supermix kit as per the manufacturer’s instructions (Bio-Rad). The primer sequences for PERK (Saito et al., 2011), IRE1α (Tsuru et al., 2016), ATF6α (Egawa et al., 2011), and 18s (Chen et al., 2009) were obtained from previous studies; and 18s was used as an internal calibrator. The experiment was performed using the Bio-Rad CFX96 real-time system (C1000 Thermal Cycler) as per the manufacturer’s guidelines. A relative fold change was analyzed by CFX Manager software (Bio-Rad) based on the ΔΔCT method. The sequences of the primers are as follows.

PERK forward: **5′- TCTTGGTTGGGTCTGATGAAT -3**’.

PERK reverse: **5′- GAT​GTT​CTT​GCT​GTA​GTG​GGG​G -3**’.

Ire1α forward: **5′-GCC​GAA​GTT​CAG​ATG​GAA​TC-3**'.

Ire1α reverse: **5′-ATC​AGC​AAA​GGC​CGA​TGA-3**'.

Atf6α forward: **5′-TTA​TCA​GCA​TAC​AGC​CTG​CG-3**’.

Atf6α reverse: **5′- CTT​GGG​ACT​TTG​AGC​CTC​TG-3**’.

18s forward primer: **5′-GGC​CCT​GTA​ATT​GGA​ATG​AGT​C-3**’.

18s reverse primer: **5′-CCA​AGA​TCC​AAC​TAC​GAG​CTT-3**’.

### Western Blotting

Wild-type (*Wt*) and *Akap1* ^*−/−*^ mice following normoxia and hyperoxia (48 h) exposure were euthanized, and the lungs were collected, snap-frozen, and stored in liquid nitrogen. The lungs were pulverized and lysed in lysis buffer (20 mM Tris–HCl, pH 7.4, 150 mM NaCl, and 0.5%, Triton X-100), and the supernatant was collected by centrifugation at 21,000 rpm for 15 min at 4°C. The amount of protein was assessed by using the BCA assay kit (Pierce, Rockford, Waltham, MA), and equal amounts of protein (5 µg) were separated by using the SDS-PAGE, followed by transfer onto PVDF membranes. The membranes were blocked in 5% BSA and washed with TBST, followed by incubation with specific primary and secondary antibodies, respectively. The primary antibodies used were BiP, JNK, *p*-JNK, eIF2α, *p*-eIF2α, Atg12, Beclin-1, CHOP (C/EBP homologous protein), Lc3b, β-actin (Cell Signaling Technology, Danvers, MA), and Erp57 (Stressgen, Farmingdale, NY), and the secondary antibodies used were anti-rabbit and anti-mouse conjugated to HRP (Jackson ImmunoResearch, West Grove, PA). The proteins were visualized by using Kwik quant ECL solution (Kindle Biosciences, Greenwich, CT) and quantified by using ImageJ (NIH, Bethesda, MD). The ratio of protein to its loading control (β-actin) was recorded. A master mix was prepared by diluting lung lysates to get a protein concentration of 1 µg/µl. From this master mix, equal amount of protein (5 µg) was separated on SDS-PAGE and probed with anti-β-Actin HRP conjugated antibody. The same set of β-Actin control image is shown in multiple figures as it represents identical set of lung lysates used for western blot analyses.

### Immunohistochemistry

After euthanizing the mice, the left lung was fixed in 4% paraformaldehyde; immunohistochemistry (IHC) was performed on paraffin-embedded lung tissue sections. In brief, the paraffin-embedded lung sections were deparaffinized with xylene, and the tissue sections were subjected to heat-induced antigen retrieval in a Tris buffer (10 mM Tris–HCl buffer at pH 9). Furthermore, endogenous peroxidase activity was quenched by incubating the sections in 3% hydrogen peroxide for 20 min, and the tissue sections were blocked with 10% goat serum for 20 min. The sections were incubated with specific primary antibodies (BiP, *p*-JNK, Erp57, and Lc3b) overnight at 4°C. The following day, the sections were probed with goat anti-rabbit antibodies (HRP-conjugated). Finally, detection of protein was carried out by using the Immpact VIP peroxidase substrate kit (Vector Laboratories, Burlingame, CA) (Fukumoto et al., 2019). The sections were imaged by using a microscope (Olympus BX43, Tokyo, Japan), attached to an Olympus DP21. The images were processed using Adobe Photoshop ver C56.

### Statistical Analysis

The data are represented as mean ± S.E.M. Statistical analysis was carried out using GraphPad Prism (ver 10, San Diego, CA). Comparisons of multiple groups utilized one-way ANOVA, followed by Tukey’s post hoc test, for normally distributed data, and *p < 0.05* was considered statistically significant.

## Results

### Genotyping and Expression of *Akap*1 Mice

The genotyping was confirmed by PCR. The agarose gel shows the *Wt* band (600 bp) and the *Akap*1 KO band (400 bp) (Supplementary Figure S1). The basal protein levels of *Akap1* were previously demonstrated by Western blot (Narala et al., 2018).

### Effect of *Akap1* Deletion on ER Stress Receptor Transcripts After Hyperoxia

**FIGURE 1:**
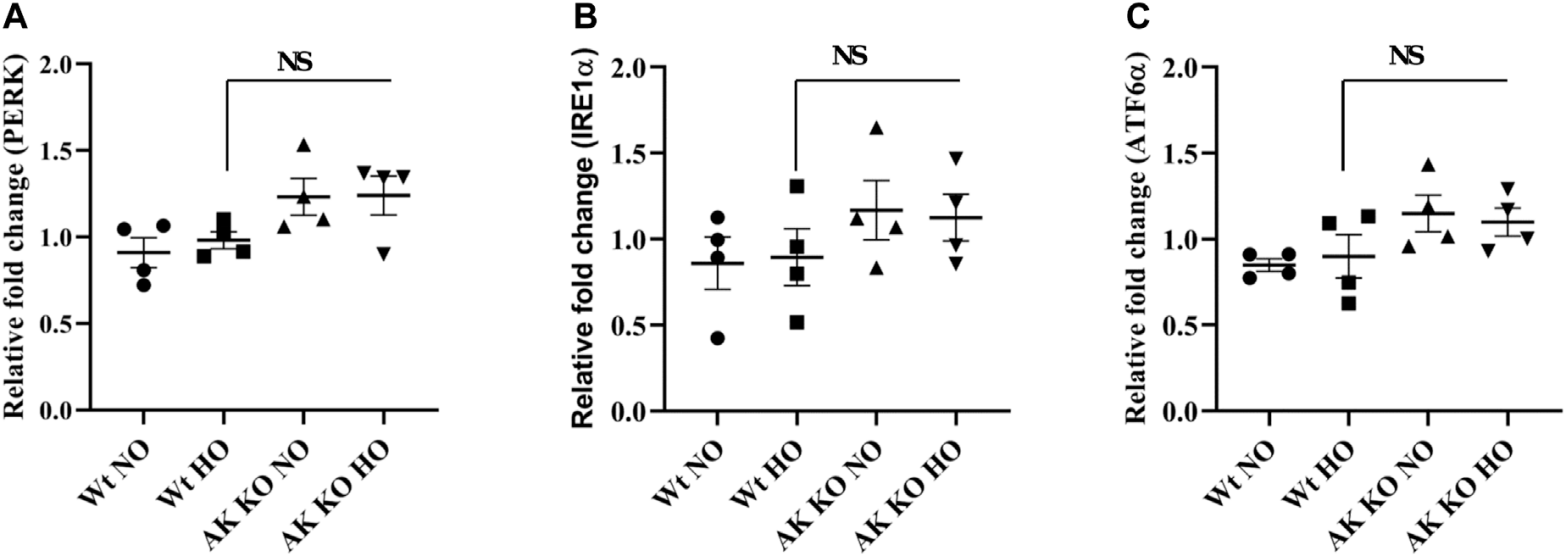
Effect of *Akap1* genetic deletion on ER stress-related receptor transcripts: *Wt* and *Akap1* mice were exposed to normoxia and hyperoxia for 48 h. After hyperoxia, the mice were euthanized, and total RNA was isolated from the lungs and subjected to qRT-PCR analysis and quantified using the ΔΔCt method. Relative fold change in **(A)** PERK, **(B)** IRE1α, and **(C)** ATF6α is shown. Data are expressed as mean ± SEM (*n* = 4 mice per group). NS, not significant; NO, normoxia; HO, hyperoxia; AK, *Akap1*; KO: Knockout.

*Wt* and *Akap1* ^*−/−*^ mice were exposed to normoxia and hyperoxia as described in earlier studies to investigate the impact of *Akap1* deletion on ER stress receptors (Kolliputi and Waxman, 2009b; Kolliputi et al., 2010). The lung samples were subjected to qRT-PCR analysis in *Akap1* ^*−/−*^ versus *Wt* mice under normoxia, and the data show a 1.35-, 1.36-, and 1.35-fold increase in PERK, IRE1α, and ATF6α, respectively (Figures 1A–C) However, no significant increase was observed under hyperoxia exposure, suggesting that *Akap1* ^*−/−*^ may cause slight upregulation of unfolded protein response (UPR) at the transcript level under normoxia.

### *Akap1* Deletion Enhances BiP Levels After Hyperoxia

**FIGURE 2:**
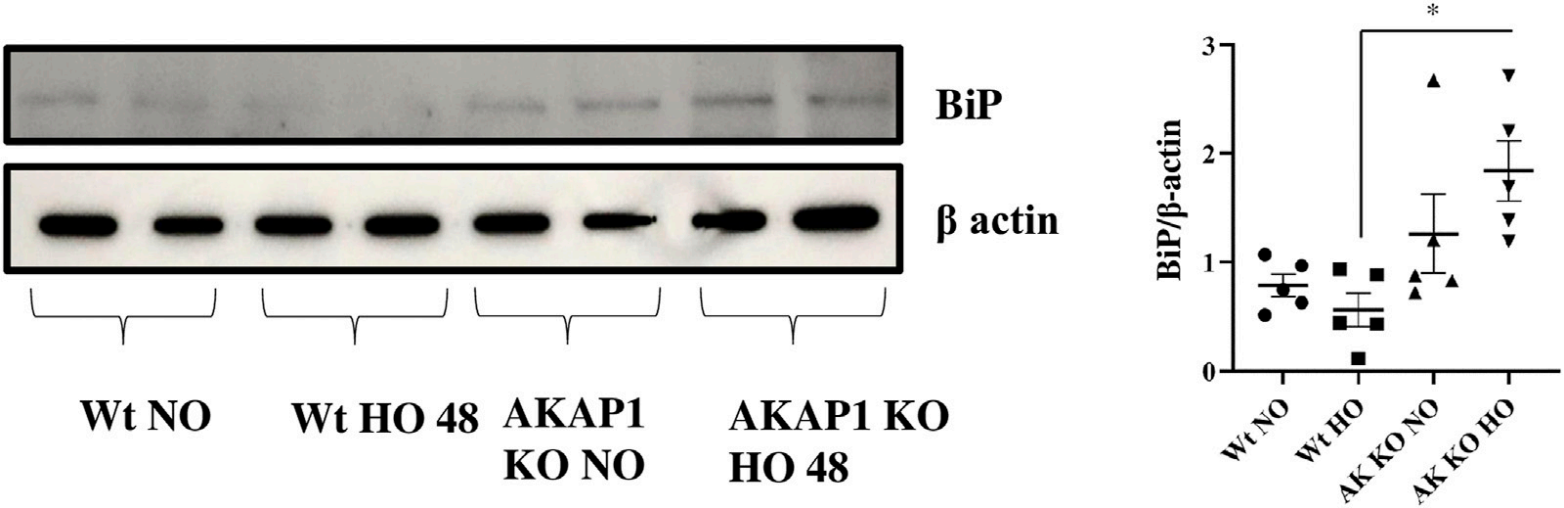
*Akap1* genetic deletion activates BiP after hyperoxic exposure: *Wt* and *Akap1* mice were exposed to normoxia and hyperoxia for 48 h. After hyperoxia, the mice were killed, and protein from the lungs was extracted and subjected to Western blot analysis to evaluate the expression of BiP, and β-actin was used as a loading control. Densitometry analysis of protein was carried out, and fold change was calculated after normalizing to β-Actin. Data are shown as mean ± S.E.M (*p < 0.05) (n = 5 mice per group), NO, normoxia; HO, hyperoxia; AK, *Akap1*; KO: Knockout.

*Wt* and *Akap1* ^*−/−*^ mice were exposed to hyperoxia or kept in normoxia to evaluate the interaction between *Akap1* deletion and BiP. A significant increase of 3.28-fold occurred in the BiP expression (Figure 2) in *Akap1* ^*−/−*^ versus *Wt* mice exposed to hyperoxia in the lung lysates subjected to Western blot analysis. There was a non-significant 1.61- and 2.25-fold increase in BiP expression in *Akap1* ^*−/−*^ mice under normoxia versus *Wt* normoxia and *Wt* hyperoxia, respectively. Enhanced BiP signals in the lung samples as evaluated by IHC were observed in the alveolar and peribronchial regions in *Akap1* ^*−/−*^ versus *Wt* mice exposed to hyperoxia (Supplementary Figures S2A,B).

### *Akap1* Deletion Activates JNK After Hyperoxia

**FIGURE 3:**
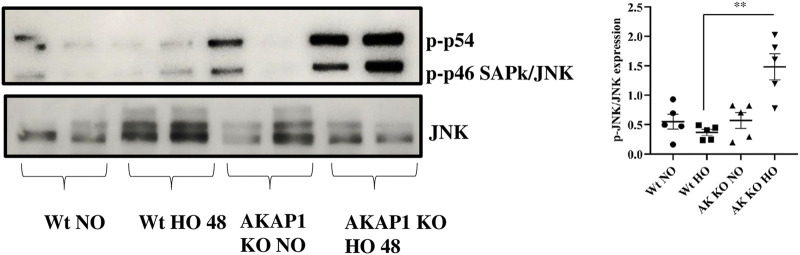
*Akap1* genetic deletion activates JNK after hyperoxic exposure: *Wt* and *Akap1* mice were exposed to normoxia and hyperoxia for 48 h. After hyperoxia, the mice were killed, and protein from the lungs was extracted and subjected to Western blot analysis to evaluate the expression of JNK and *p*-JNK. Densitometry analysis was carried out, and after normalization with β-actin, the results were expressed in fold change. Data are shown as mean ± S.E.M (**p < 0.01) (*n* = 5 mice per group), NO, normoxia; HO, hyperoxia; AK, *Akap1*; KO: Knockout.

*Wt* and *Akap1* ^*−/−*^ mice were exposed to normoxia or hyperoxia to investigate the relationship between *Akap1* deletion and JNK. The immunoblot analysis showed a 4.1-fold significant increase in the expression of the *p*-JNK (Figure 3) in *Akap1* ^*−/−*^ versus *Wt* mice exposed to hyperoxia. There was no difference under normoxia between *Wt* and *Akap1* ^*−/−*^ mice. The expression of *p*-JNK was evaluated by IHC in the lung samples. Increased *p*-JNK signals (Supplementary Figure.S3) were observed in the peribronchial region of *Akap1* ^*−/−*^ versus *Wt* mice exposed to hyperoxia.

### *Akap1* Deletion Causes Phosphorylation of Eukaryotic Initiation Factor

**FIGURE 4:**
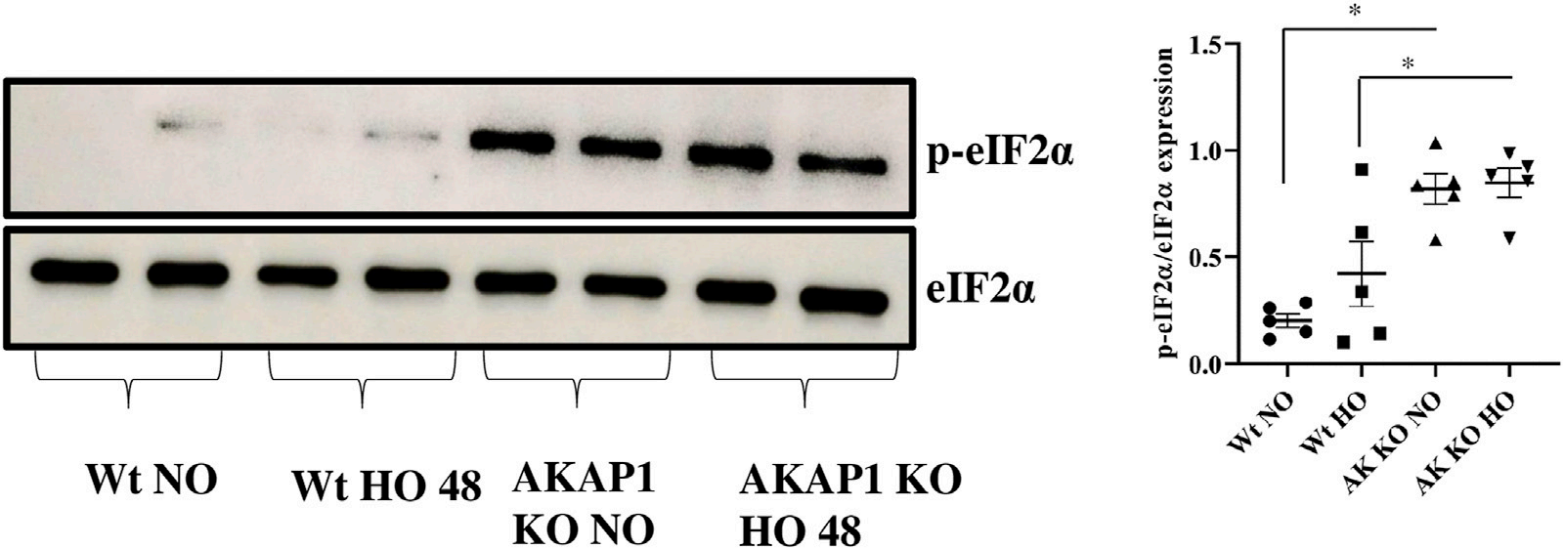
*Akap1* genetic deletion causes phosphorylation of eukaryotic initiation factor (eIF2α): *Wt* and *Akap1* mice were exposed to normoxia and hyperoxia for 48 h. After hyperoxia, the mice were killed, and protein was extracted from the lungs and subjected to Western blot analysis. Following densitometry analysis and normalization with β-actin, the ratio of *p*-eIF2α to eIF2α protein levels was evaluated. Results are expressed in fold change. Data are shown as mean ± SEM (*p < 0.05) (*n* = 5 mice per group). NO, normoxia; HO, hyperoxia; AK, *Akap1*; KO: Knockout.

*Wt* and *Akap1* ^*−/−*^ mice were exposed to normoxia or hyperoxia to investigate the interaction between *Akap1* deletion and eIF2α. Immunoblot results showed a 4-fold significant increase in the expression of *p*-eIF2α (Figure 4) in *Akap1* ^*−/−*^ versus *Wt* mice in normoxia and a 2-fold significant increase in *Akap1* ^*−/−*^ versus *Wt* following hyperoxia.

### *Akap1* Deletion Causes ER Stress-Induced Cell Death After Hyperoxia

**FIGURE 5:**
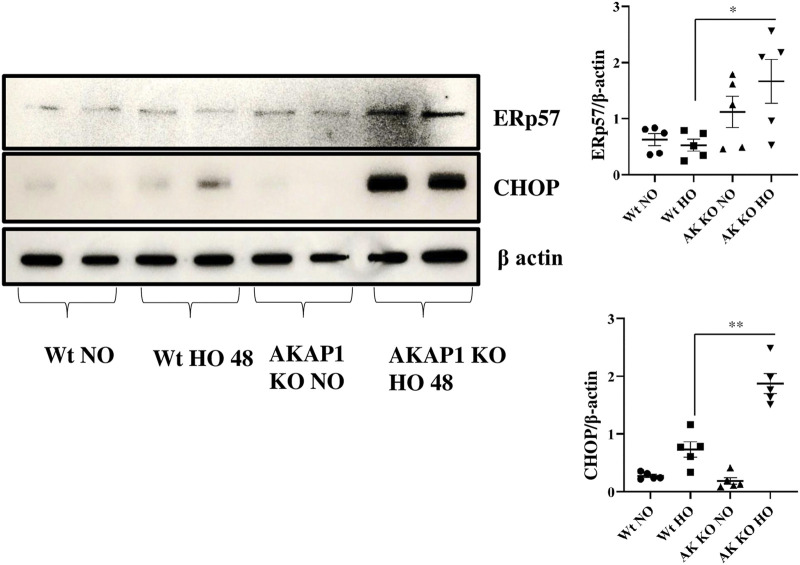
*Akap1* genetic deletion enhances ER stress-induced cell death after hyperoxic exposure: *Wt* and *Akap1* mice were exposed to normoxia and hyperoxia for 48 h. After hyperoxia, the mice were killed, and protein was extracted from the lungs and subjected to Western blot analysis to evaluate the expression of CHOP and Erp57. β-actin was used as a loading control. Densitometry analysis of CHOP and Erp57 protein was followed by normalization to β-actin. Data are expressed as mean ± SEM (*p < 0.05, **p < 0.005) (*n* = 5 mice per group). NO, normoxia; HO, hyperoxia; AK, *Akap1*; KO: Knockout.

*Wt* and *Akap1* ^*−/−*^ mice were exposed to normoxia or hyperoxia to investigate the connection between *Akap1* deletion and ER stress-induced cell death. Western blot analysis indicated that there were significant increases in the expression of ERp57 (3.13-fold) and CHOP (2.56-fold) (Figure 5) in *Akap1* ^*−/−*^ versus *Wt* mice exposed to hyperoxia for 48 h. Furthermore, mouse lung tissue samples were subjected to IHC to confirm ER stress-induced cell death. ERp57 signals (Supplementary Figures S4A,B) were more abundant in the alveolar and peribronchial regions of *Akap1* ^*−/−*^ versus *Wt* mice exposed to hyperoxia for 48 h.

### *Akap1* Deletion Causes Autophagy After Hyperoxia

**FIGURE 6:**
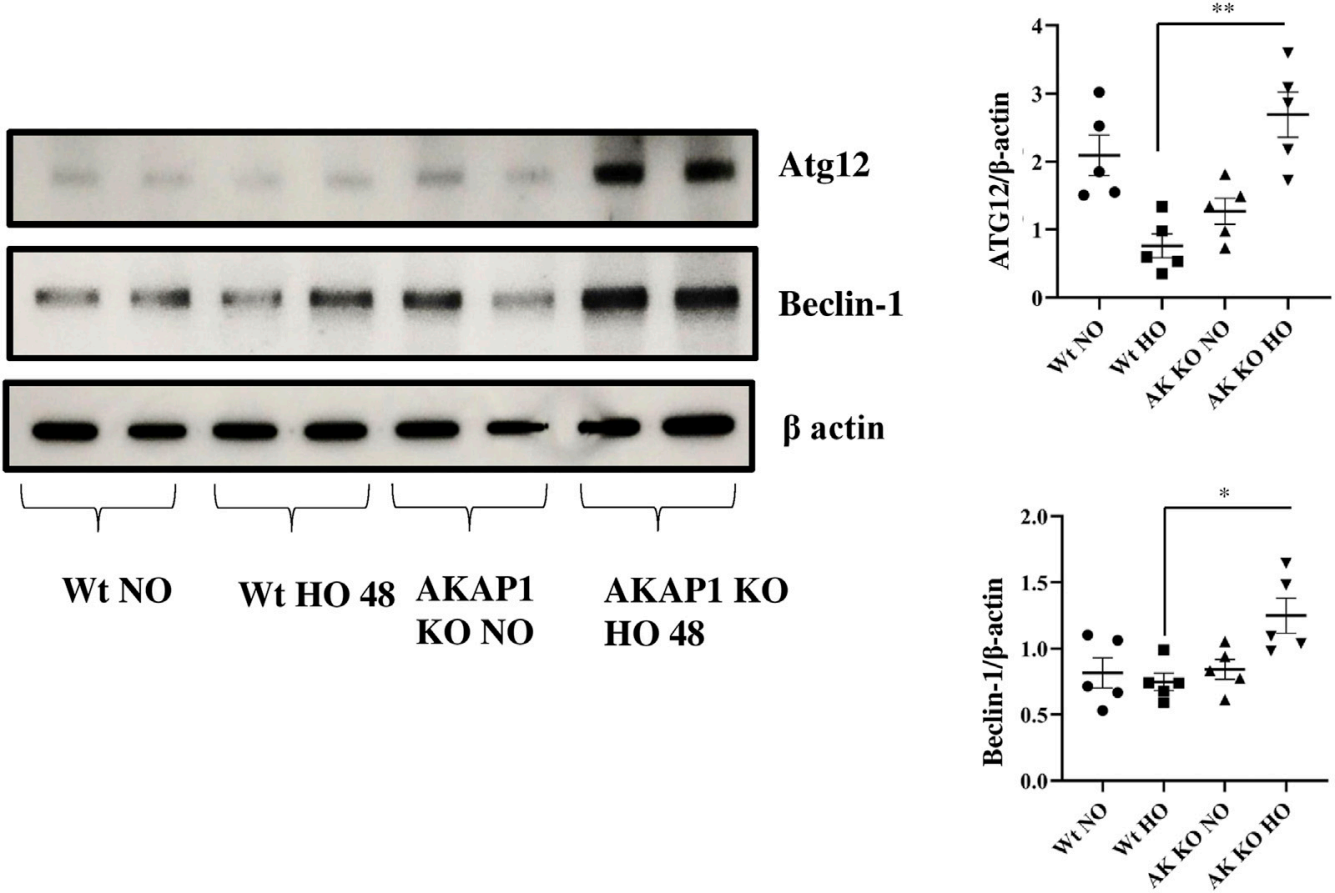
*Akap1* genetic deletion enhances autophagy after hyperoxic exposure: *Wt* and *Akap1* mice were exposed to normoxia and hyperoxia for 48 h. After hyperoxia, the mice were killed, and protein was extracted from the lungs and subjected to Western blot analysis to evaluate the protein expression of Atg12 and Beclin-1. β-actin was used as a loading control. Densitometry analysis of Atg12 and Beclin-1 was followed by normalization with β-actin. Data are shown as mean ± S.E.M (*p < 0.05, **p < 0.01) (*n* = 5 mice per group). NO, normoxia; HO, hyperoxia; AK, *Akap1*; KO: Knockout.

The protein autophagy-related gene 12 (ATG12) plays a key role in the formation of the autophagosome (Otomo et al., 2013), and the Beclin-1 and Lc3b proteins induce autophagy (Wirawan et al., 2012; Satyavarapu et al., 2018; Vega-Rubín-de-Celis, 2019). *Wt* and *Akap1* ^*−/−*^ mice were exposed to normoxia or hyperoxia to investigate the effect of *Akap1* deletion on autophagy. Immunoblot results indicate a significant 3.42- and 1.67-fold increase in the expression of ATG12 and Beclin-1 (Figure 6), respectively, in *Akap1* ^*−/−*^ versus *Wt* mice exposed to hyperoxia for 48 h. Mouse tissue samples were subjected to IHC analysis to investigate the effects on autophagy. The Lc3b (Supplementary Figures S5A,B) was highly expressed in the alveolar and peribronchial regions of *Akap1* ^*−/−*^ versus *Wt* mice exposed to hyperoxia for 48 h.

## Discussion

These studies indicate that *Akap1* genetic deletion exacerbates ER stress associated with hyperoxia. *Akap1* deletion results in the following: 1) partial activation of the ER stress receptors under normoxia, 2) BiP activation after hyperoxic exposure, 3) JNK phosphorylation following hyperoxic exposure, 4) phosphorylation of eukaryotic initiation factor, 5) an increase in ER stress-induced cell death, and 6) increased autophagy.

The ER plays an important role in the homeostasis of cells by regulating lipid synthesis, protein secretion, calcium homeostasis, and protein folding (Szegezdi et al., 2006). Disturbance in the homeostasis of the ER triggers UPR by activating the ER receptors PERK, IRE1α, and ATF6 (Malhotra and Kaufman, 2007). In this study, *Akap1* deletion shows a minimal increase in UPR receptor proteins versus *Wt* mice under normoxia. In this study, the mice were exposed to hyperoxia for 48 h which did not seem to affect the ER stress receptors (Figures 1A–C). This corroborates with another study which showed that hyperoxia does not impact ER receptors in *Wt* mice exposed to hyperoxia for 64 h (Gewandter et al., 2009). A short period of hyperoxia exposure (3–6 h) may be required to observe the effect of hyperoxia on ER stress receptor transcripts.

BiP is a hallmark of ER stress response and UPR (Ron and Walter, 2007). The consistent protein aggregation from ER stress causes a transition from pro-survival to pro-apoptotic/ER stress-induced cell death (Lu et al., 2015). Increased BiP levels were found in *Akap1* ^*−/−*^ versus *Wt* mice exposed to normoxia and hyperoxia, indicating that *Akap1* ^*−/−*^ mice are susceptible to ER stress and that hyperoxia exacerbates this stress by causing the aggregation of misfolded proteins (Lu et al., 2015). BiP expression can be increased in cancer, drug-resistant cancer cells, and dormant cancer cells (Li et al., 2011).

PKA protects cells from ER stress and *Akap*1 regulates PKA (Gewandter et al., 2009; Aguileta et al., 2016). The presence of PKA negatively regulates the JNK protein (Zeitlin et al., 2011). JNK, as with oxidative stress, environmental stress, and the presence of pro-inflammatory cytokines, is phosphorylated under stress conditions (Yamasaki et al., 2017). Our data indicate that *Akap1* deletion depletes PKA levels and elevates the phosphorylation of JNK. Hyperoxia treatment also augments *p*-JNK levels, in agreement with our previous studies (Kolliputi and Waxman, 2009b; Galam et al., 2016). Additionally, hydrogen peroxide treatment also activates JNK for initiating apoptosis (Kolliputi and Waxman, 2009b; Wei et al., 2010). JNK phosphorylation can occur during mitochondrial dysfunction, atherosclerosis, and metabolic diseases (Sano and Reed, 2013).

Prolonged hyperoxia exposure cause UPR and can induce eIF2α phosphorylation under stress conditions (Konsavage et al., 2012; Lu et al., 2015). The eIF2α phosphorylation also inhibits protein translation to induce endoplasmic stress-associated degradation (ERAD) (Ohno, 2014). *Akap1* is predominantly a mitochondrial protein. The shorter form of *Akap1* (N0) is known to target mitochondria, while the longer form (N1) targets the ER (Konsavage et al., 2010). It is plausible that the ER effects due to the *Akap1* deletion may be attributed to the N1 form. As previously observed, deletion of *Akap1* alters the size and structure of mitochondria (Schiattarella et al., 2016; Narala et al., 2018; Schiattarella et al., 2018). eIF2α phosphorylation is seen in Parkinson’s disease and Alzheimer’s disease (Sano and Reed, 2013; Ohno, 2014). These results suggest that *Akap1* deletion leads to eIF2α phosphorylation under normoxic and hyperoxic conditions. Further studies are required to evaluate the interaction between *Akap1* and eIF2α phosphorylation in human cell lines.

CHOP is expressed during ER stress and can lead to apoptosis (Xu et al., 2009; Hoffman et al., 2016). This protein is usually present at low levels but is upregulated and activated by eIF2α phosphorylation during ER stress, which causes DNA damage and growth arrest (Oyadomari and Mori, 2004; Xu et al., 2009; Hoffman et al., 2016). The protein, ERp57, is also expressed during ER stress and causes apoptosis (Xu et al., 2009; Hoffman et al., 2016). The data show an increase in ER stress-induced cell death markers, CHOP and ERp57, in *Akap1* ^*−/−*^ versus *Wt* mice exposed to hyperoxia. CHOP expression was seen in viral infection, neurodegenerative disease, atherosclerosis, metabolic disease, inflammation, and ophthalmology disease (Sano and Reed, 2013). The *Akap1* ^*−/−*^ mice under normoxia have increased expression of eIF2α phosphorylation but do not undergo CHOP mediated cell death. *Akap1* ^*−/−*^ mice under normoxia display enhanced ERp57 expression. It is possible that *Akap1* ^*−/−*^ mice may suffer ERp57-mediated cell death *via* the NFκB or STAT3 pathway (Liu et al., 2019). A wide array of evidence shows that ERp57 dysregulation occurs in melanoma, laryngeal cancer, and leukemia (Liu et al., 2019). ERp57 deletion causes a decrease in inflammatory cells, epidermal growth factor, periostin, and increased airway resistance (Hoffman et al., 2016).

ER stress leads to cellular degradation, apoptosis, inflammation, autophagy, mitophagy, and protein degradation (Senft and Ronai, 2015). The protein ATG12 plays a role in autophagy initiation and causes mitochondrial apoptosis (Rubinstein et al., 2011), while Beclin-1 is a regulator of autophagy, and LC3b is a marker of autophagy (Meyer et al., 2013). The data suggest that *Akap1* deletion induces autophagy compared to *Wt* mice exposed to hyperoxia. The expression of ATG12 in *Wt* mice is higher than that of *Akap1* ^*−/−*^ mice under normoxia. This suggests that certain cells undergo the formation of autophagosomes to maintain mitochondrial homeostasis due to damaged mitochondria (Radoshevich et al., 2010). Also, *Lc3b* ^−/-^ mice demonstrate decreased caspase activity (Sauler et al., 2015). Cho et al., 2009 found that Beclin-1 links apoptosis to autophagy in HELA cells mediated by caspases (Cho et al., 2009). Autophagy is also associated with neurodegenerative disease and cancer promotion, suggesting the impact of *Akap1* ^*−/−*^ on autophagy (Sano and Reed, 2013).

These findings suggest that *Akap1* has a crucial role in the lung in the setting of hyperoxic exposure. *Akap1*-knockout mice exposed to hyperoxia show activation of BiP, JNK phosphorylation, ER stress-induced cell death, autophagy, and eIF2α phosphorylation (even under normoxia). Therefore, *Akap1* is a potential therapeutic target in ALI for patients who require supplemental oxygen.

## Data Availability

The raw data supporting the conclusions of this article will be made available by the authors, without undue reservation.

## Abbreviations

AK, Akap1; AV, alveoli; HO, hyperoxia; KO, knockout; NO, normoxia; PB, peribronchial; Wt, wild type.
