# Supplementary figures and images for: Mitochondrial Protein Akap1 Deletion Exacerbates Endoplasmic Reticulum Stress in Mice Exposed to Hyperoxia
Source: Front Pharmacol. 2022 Mar 14;13:762840. doi: 10.3389/fphar.2022.762840 (PMC8964370; doi:10.3389/fphar.2022.762840)

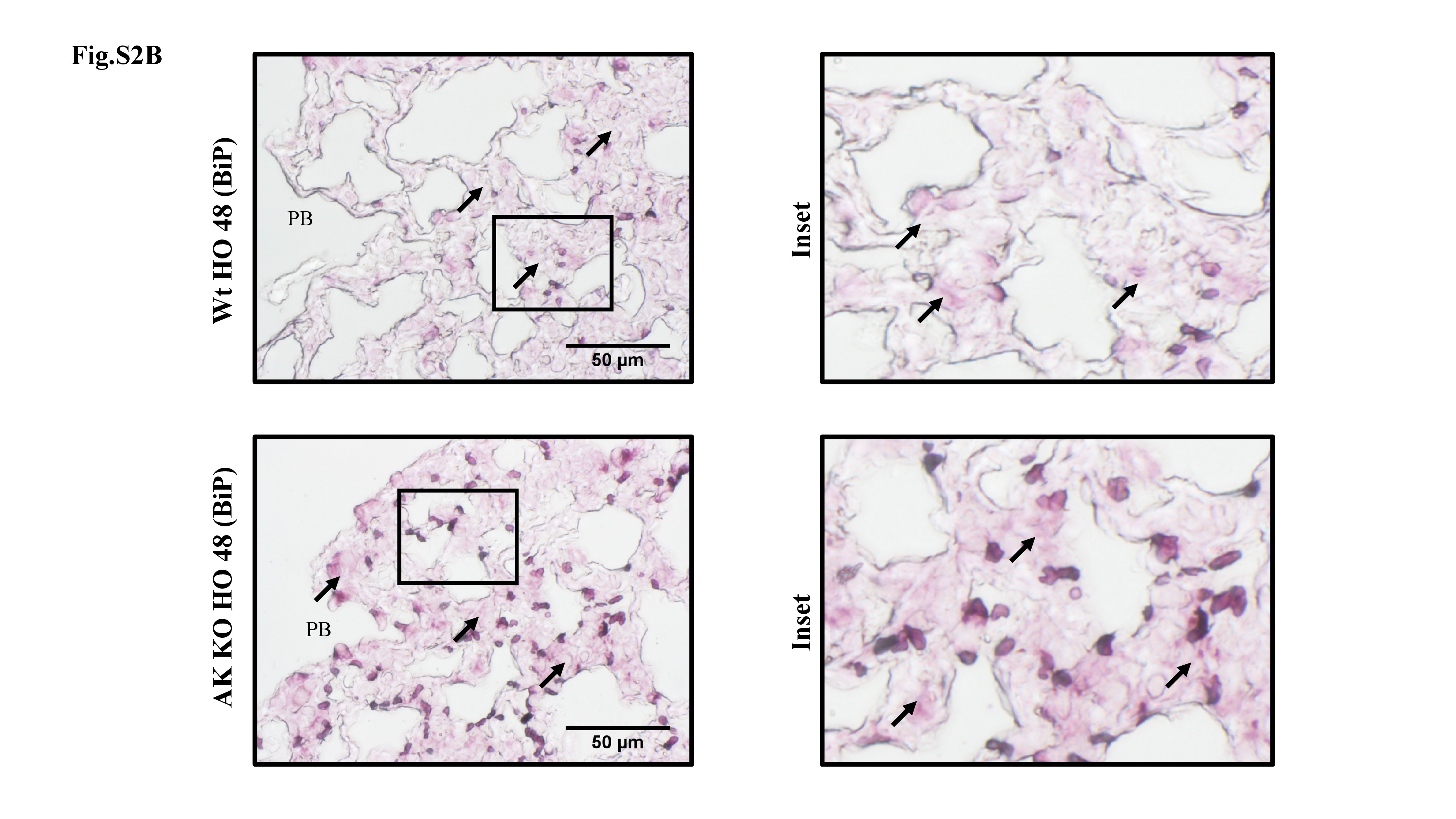

Supplement: Supplementary file 1 [file Image3.JPEG]

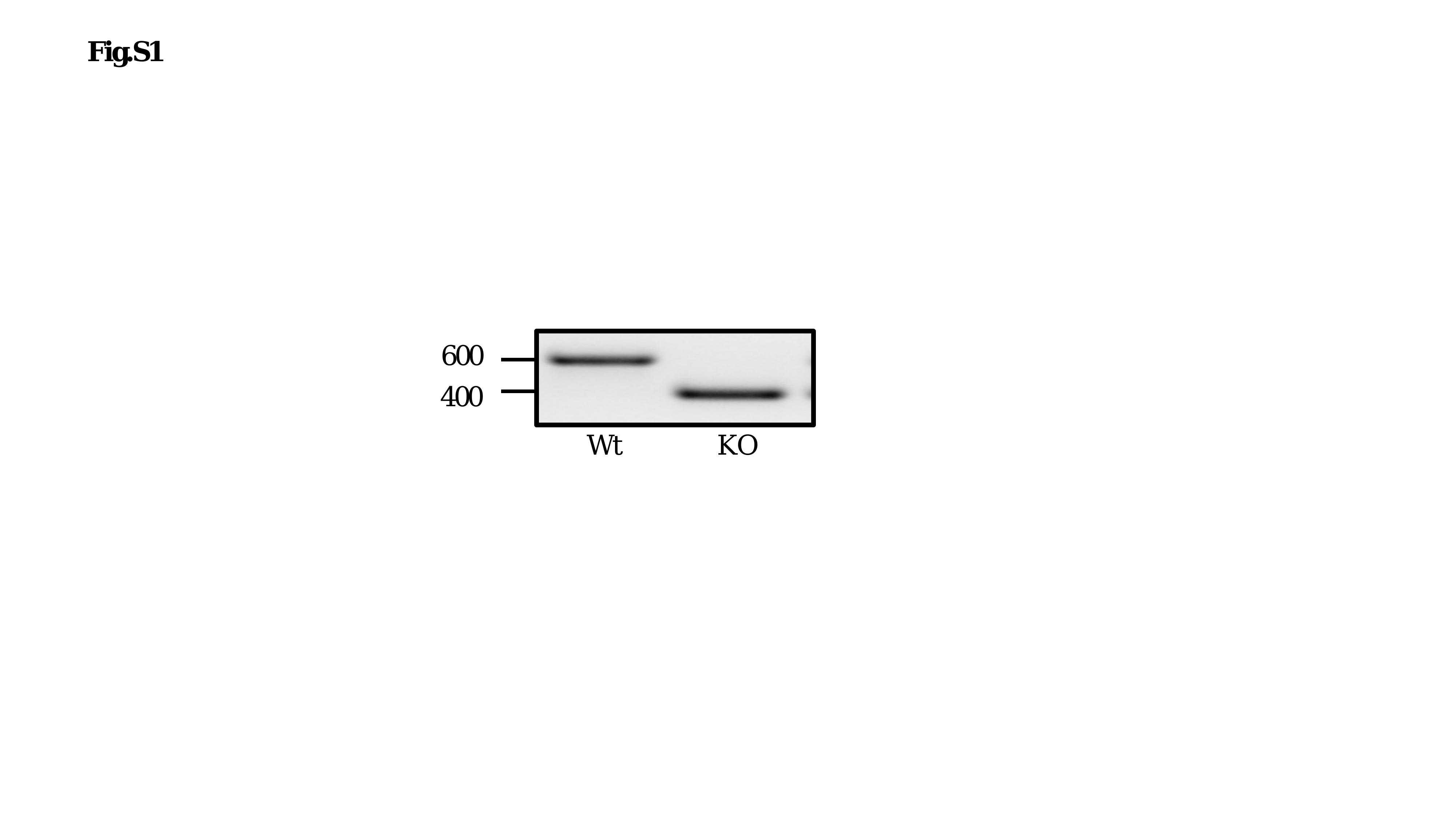

Supplement: Supplementary file 2 [file Image1.JPEG]

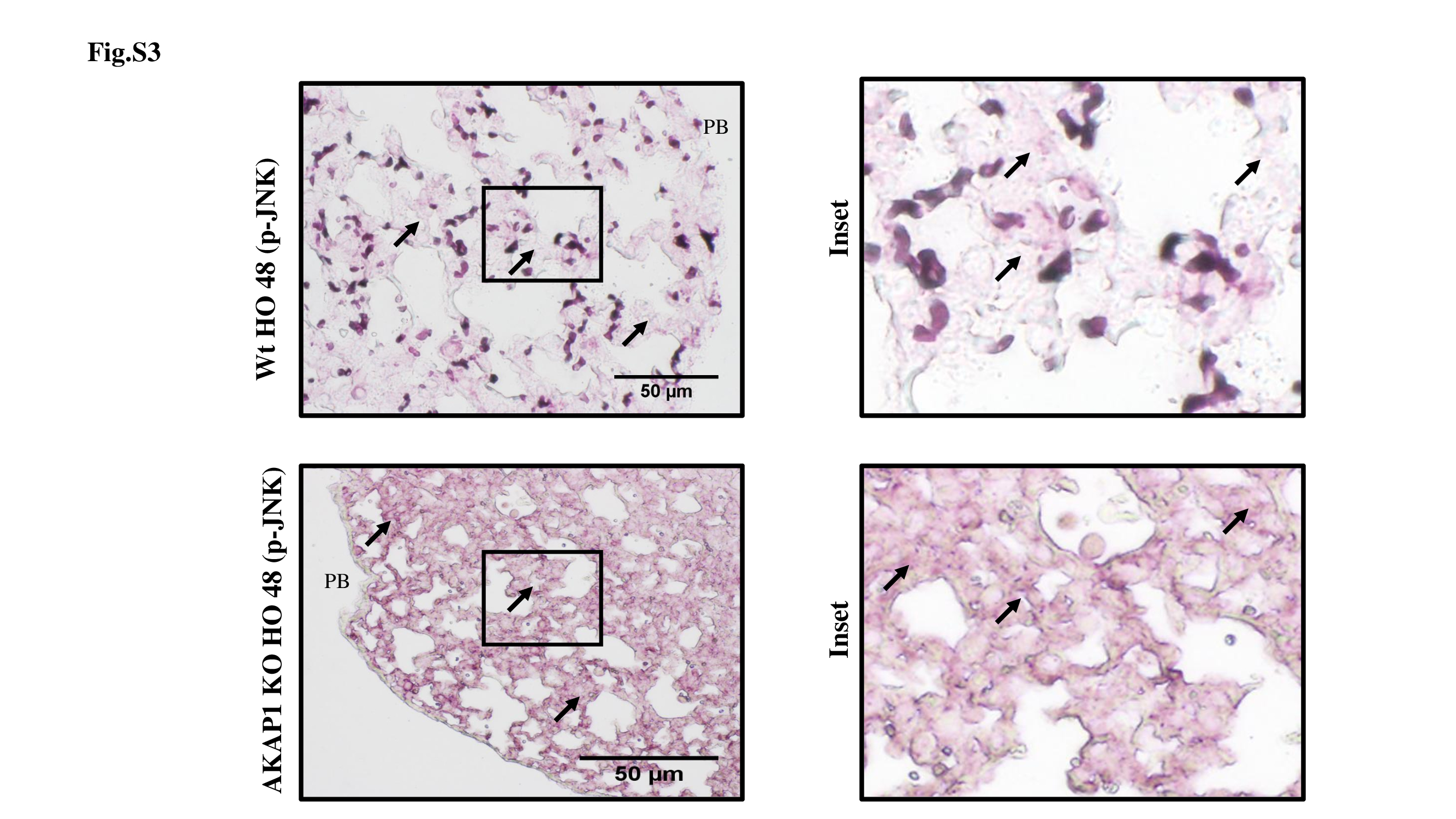

Supplement: Supplementary file 3 [file Image4.JPEG]

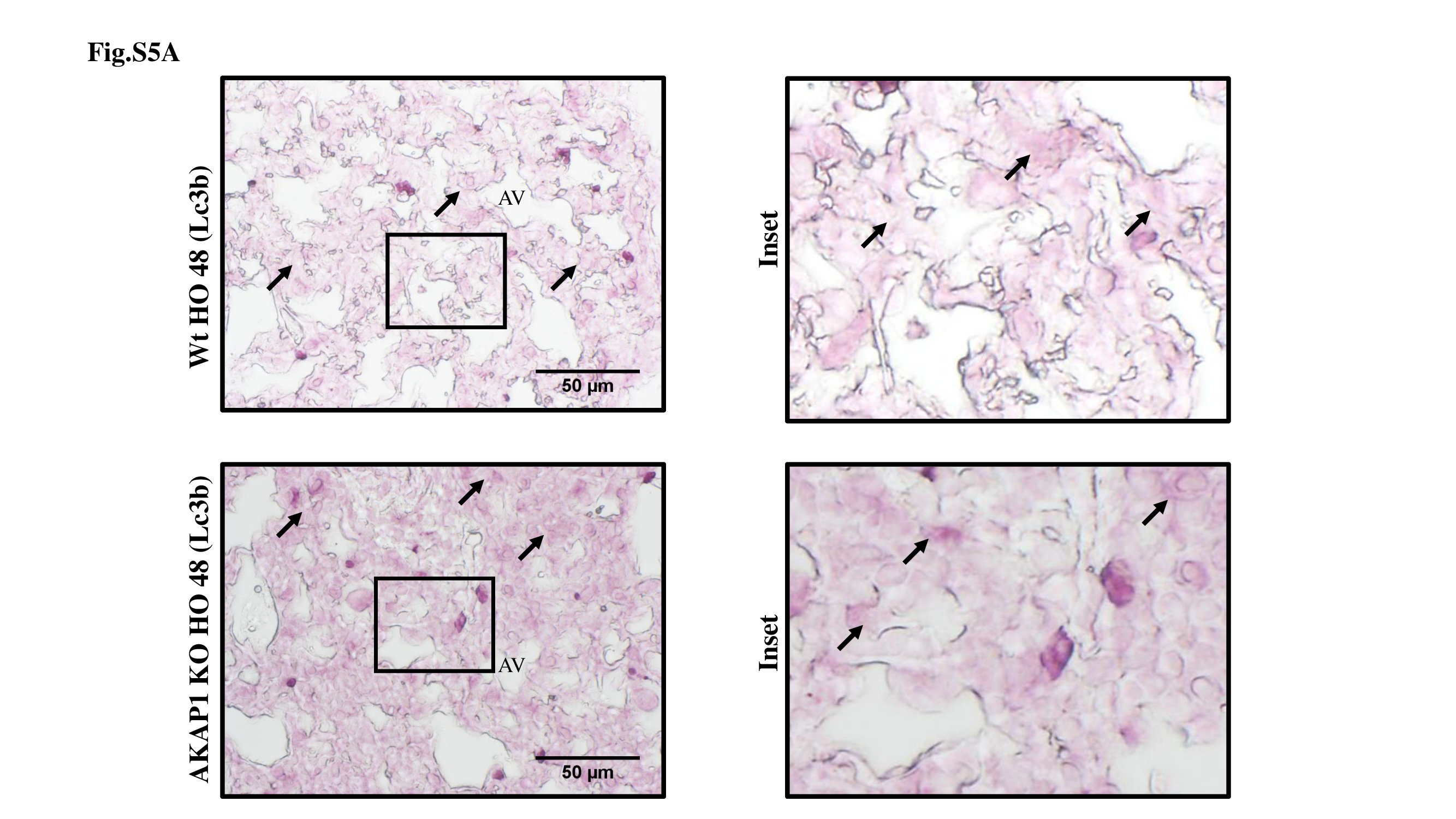

Supplement: Supplementary file 4 [file Image7.JPEG]

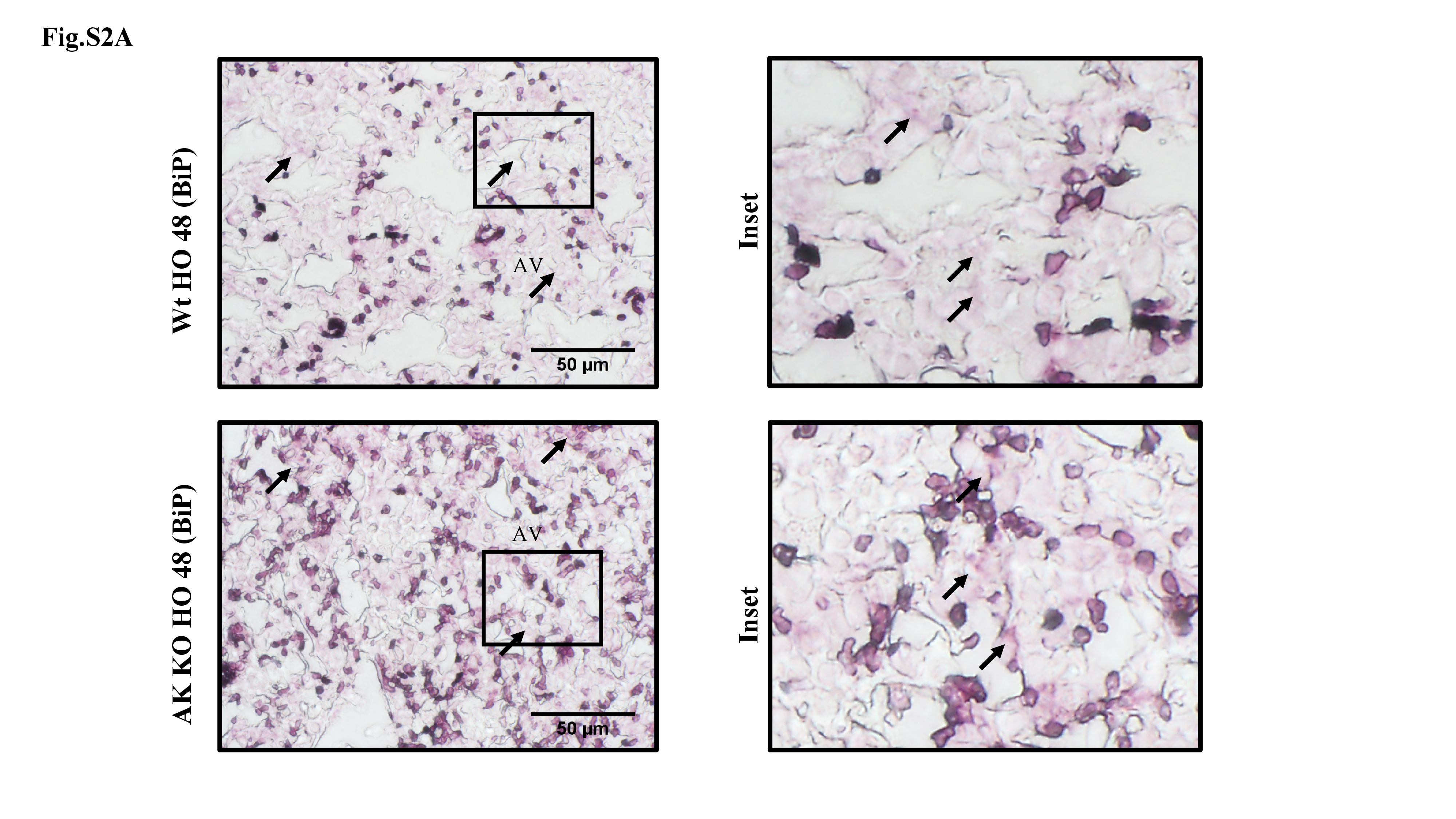

Supplement: Supplementary file 5 [file Image2.JPEG]

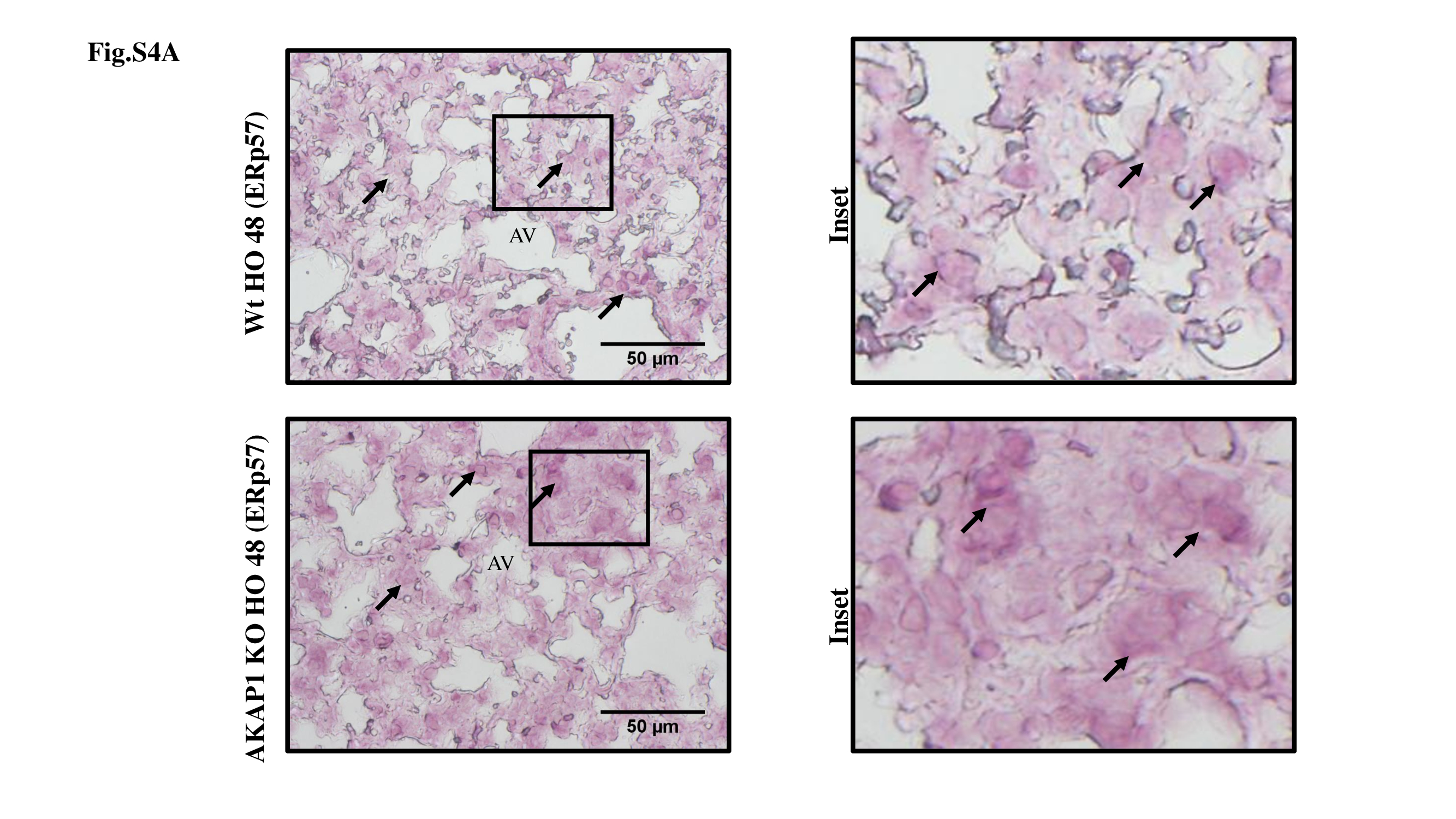

Supplement: Supplementary file 6 [file Image5.JPEG]

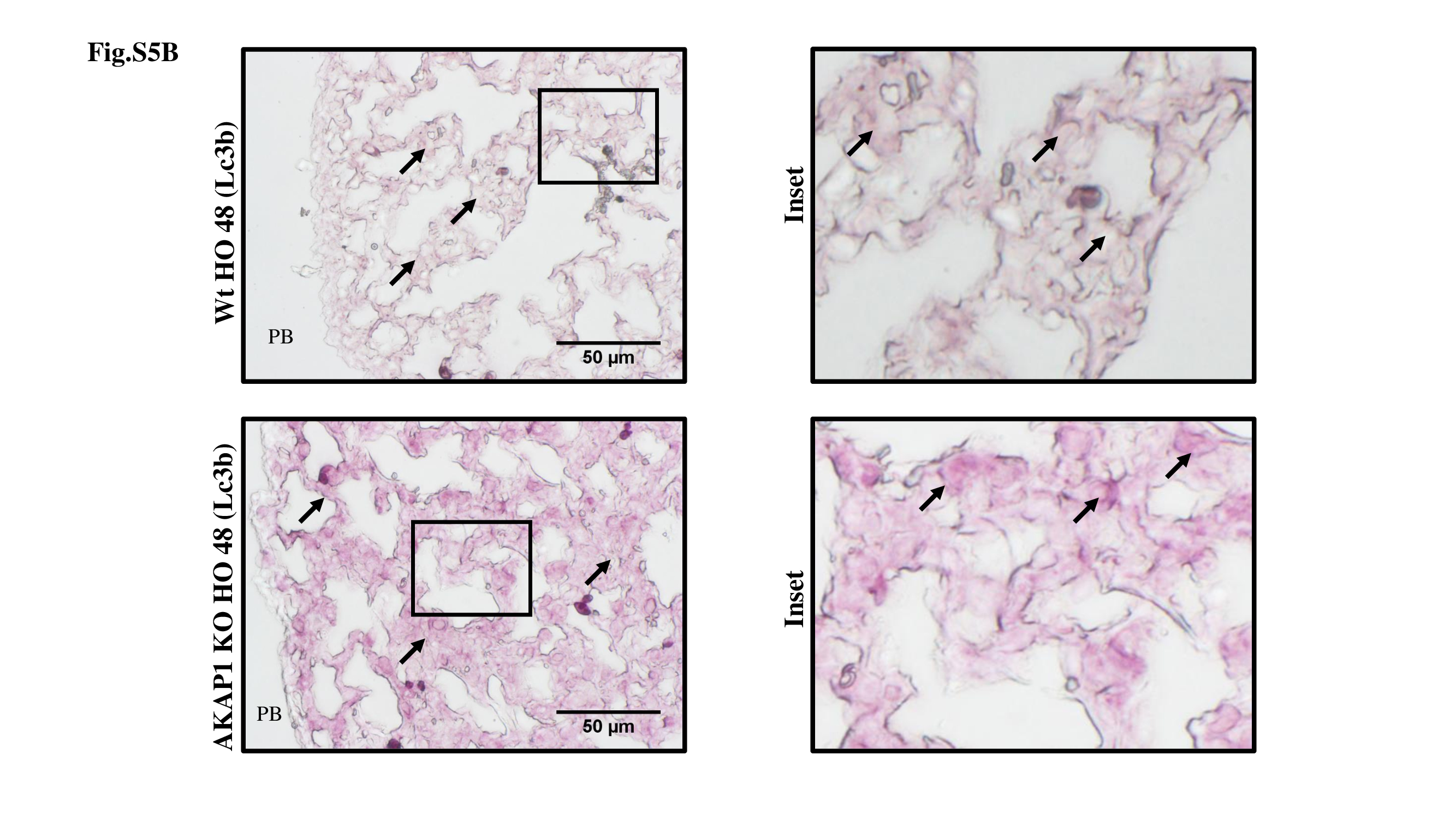

Supplement: Supplementary file 8 [file Image8.JPEG]

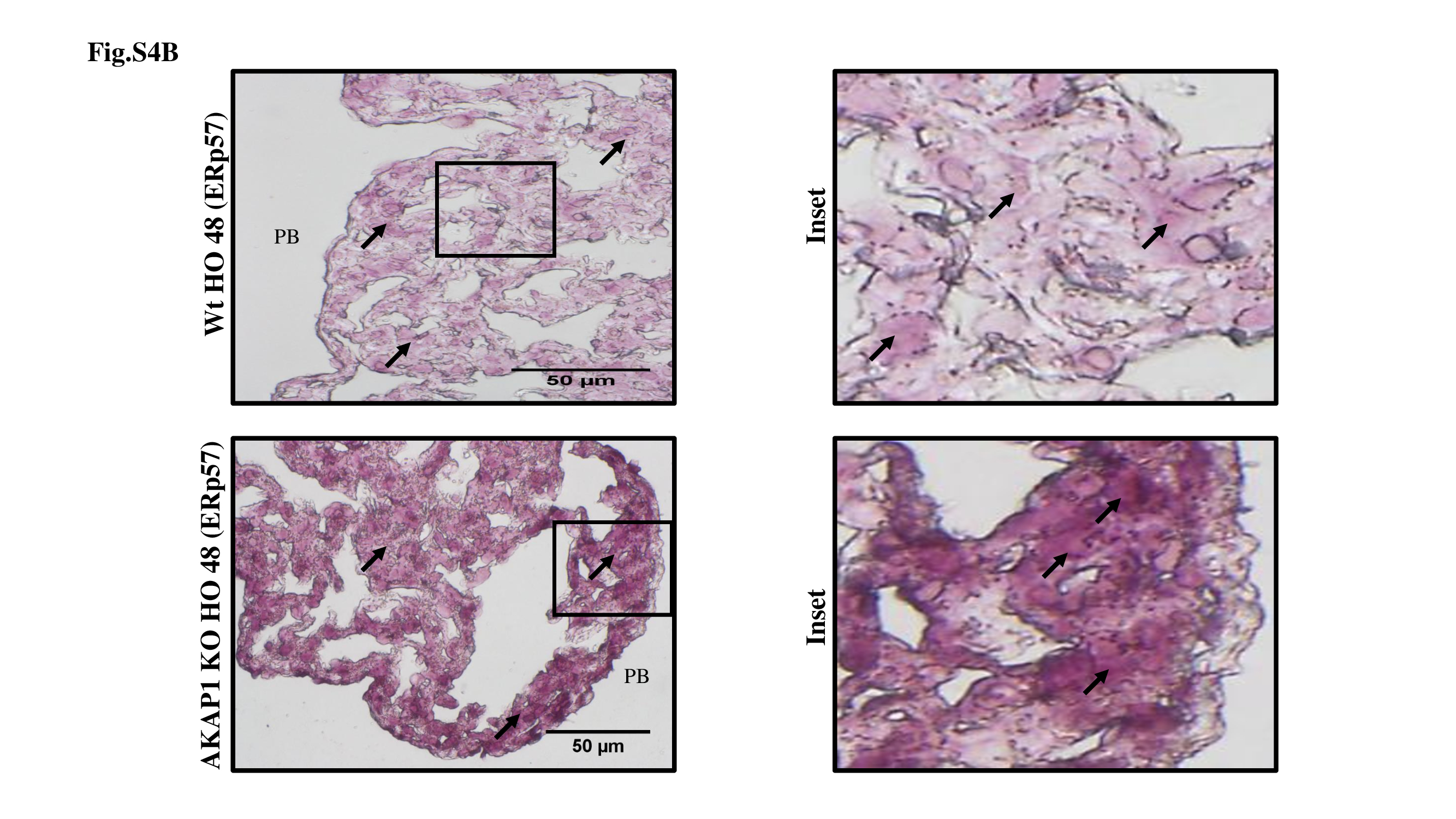

Supplement: Supplementary file 9 [file Image6.JPEG]
