# Supplementary material for: Mitochondrial Protein Akap1 Deletion Exacerbates Endoplasmic Reticulum Stress in Mice Exposed to Hyperoxia
Source: Front Pharmacol. 2022 Mar 14;13:762840. doi: 10.3389/fphar.2022.762840 (PMC8964370; doi:10.3389/fphar.2022.762840)
Supplement: Supplementary file 7 [file DataSheet1.PDF]

Supplemental Figures

Fig.S1. *Akap1* genotyping: Genomic DNA was extracted from the *Wt* and *Akap1*$^{-/-}$ mice and PCR was performed to determine the genotype.

Fig. S2A and S2B. Akap1 genetic deletion mice exposed to hyperoxia show enhanced BiP signal: Immunohistochemical analysis of BiP on lung sections derived from *Wt* and *Akap1*$^{-/-}$ mice exposed to hyperoxia for 48 h. Microscopic evaluation of BiP staining in *Wt* and *Akap1*$^{-/-}$ mice exposed to hyperoxia at 400x (n=3 mice per group) (AV: Alveolar, PB: Peri-bronchial area) (Arrow indicates the amount of stain and inset are enlargement of the selected area).

Fig. S3. Akap1 genetic deletion mice exposed to hyperoxia show enhanced p-JNK signal: Immunohistochemical staining of p-JNK is performed on lung sections from *Wt* and *Akap1*$^{-/-}$ mice exposed to hyperoxia for 48 h. Microscopic evaluation of *Wt* and *Akap1*$^{-/-}$ mice exposed to hyperoxia at 400x (n=3 mice per group) (PB: Peri-bronchial area) (Arrow indicates the amount of stain and inset are enlargement of selected area).

Fig. S4A and S4B. Akap1 genetic deletion mice exposed to hyperoxia show enhanced ERp57 signal: Immunohistochemical staining of Erp57 was performed on lung sections from *Wt* and *Akap1*$^{-/-}$ mice exposed to hyperoxia for 48 h. Microscopic evaluation of *Wt* and *Akap1*$^{-/-}$ mice exposed to hyperoxia at 400x (n=3 mice per group) (AV: Alveolar, PB: Peri-bronchial area) (Arrow indicates the amount of stain and inset are enlargement of the selected area).

Fig. S5A and S5B. Akap1 genetic deletion mice exposed to hyperoxia show enhanced Lc3b signal: Immunohistochemical staining of Lc3b was performed on lung sections from *Wt* and *Akap*1$^{-/-}$ mice exposed to hyperoxia for 48 h. Microscopic evaluation of *Wt* type and *Akap1*$^{-/-}$

mice exposed to hyperoxia 400x (n=3 mice per group) (AV: Alveolar, PB: Peri-bronchial area) (Arrow indicates the amount of stain and inset are enlargement of selected area).
